# Laser Teeth Bleaching: Evaluation of Eventual Side Effects on Enamel and the Pulp and the Efficiency In Vitro and In Vivo

**DOI:** 10.1155/2015/835405

**Published:** 2015-03-22

**Authors:** Roeland Jozef Gentil De Moor, Jeroen Verheyen, Peter Verheyen, Andrii Diachuk, Maarten August Meire, Peter Jozef De Coster, Mieke De Bruyne, Filip Keulemans

**Affiliations:** ^1^Department of Restorative Dentistry and Endodontology, Ghent Dental Laser Centre, Ghent Dental Photonics Research Cluster, Ghent University, Ghent University Hospital, Dental School, De Pintelaan 185-P8, 9000 Gent, Belgium; ^2^Department of Clinical Neurosciences, John van Geest Centre for Brain Repair and Wellcome Trust-Medical Research Council Stem Cell Institute, University of Cambridge, Clifford Allbutt Building, Cambridge Biosciences Campus, Cambridge, CB2 0QH, UK; ^3^SOLA Academy, Bernhard Gottlieb University Clinic of Dentistry, Sensengasse 2A, 1090 Vienna, Austria

## Abstract

Light and heat increase the reactivity of hydrogen peroxide. There is no evidence that light activation (power bleaching with high-intensity light) results in a more effective bleaching with a longer lasting effect with high concentrated hydrogen peroxide bleaching gels. Laser light differs from conventional light as it requires a laser-target interaction. The interaction takes place in the first instance in the bleaching gel. The second interaction has to be induced in the tooth, more specifically in the dentine. There is evidence that interaction exists with the bleaching gel: photothermal, photocatalytical, and photochemical interactions are described. The reactivity of the gel is increased by adding photocatalyst of photosensitizers. Direct and effective photobleaching, that is, a direct interaction with the colour molecules in the dentine, however, is only possible with the argon (488 and 415 nm) and KTP laser (532 nm). A number of risks have been described such as heat generation. Nd:YAG and especially high power diode lasers present a risk with intrapulpal temperature elevation up to 22°C. Hypersensitivity is regularly encountered, being it of temporary occurrence except for a number of diode wavelengths and the Nd:YAG. The tooth surface remains intact after laser bleaching. At present, KTP laser is the most efficient dental bleaching wavelength.

## 1. Introduction

Heating hydrogen peroxide (HP) results in an acceleration of its decomposition and oxidant-free radical formation [1]. Therefore, the dental bleaching process can be accelerated by additional heat activation. One of the activation methods resulting in an increase of the temperature in the bleaching gel is power bleaching with high-intensity light [2].

The effectiveness of this method for vital tooth bleaching has been demonstrated in animal studies, clinical studies and reports, and a number of reviews [3–6]. Side effects for the tooth, that is, alteration of the enamel surface, posttreatment, and pulp sensitivity, have been suggested and investigated [4, 5, 7, 8].

Potential adverse effects on enamel were primarily investigated in vitro using extracted human and bovine teeth. Reports on the effects of light-activated systems were divergent, which was also the case for conventional in-office bleaching techniques. On the one hand, changes in microhardness, the presence of porosities, changes in surface roughness, a reduction in fracture toughness, alteration of the calcium/phosphate ratio, erosion, decrease in abrasion resistance, and the formation of depressions were reported. The enamel surface changes varied mostly with the bleaching products used, especially high concentrations of hydrogen peroxide; that is, 30–35% (w/w) and 35% (w/w) carbamide peroxide (CPO) (11-12% HP) could have a damaging effect, whereas low concentrations 10% or 16% CPO (w/w) (3–5% HP) had no effect [8]. On the other hand, rehardening of porous enamel as a result of saliva ion reprecipitation has been described. Although remineralisation due to the saliva may be responsible for a gradual mineral rebuild-up, full repair of the enamel is not established due to a degradation of the organic matrix [8]. To date, nevertheless, no clinical adverse effects of power bleaching on enamel have been reported.

Sensitivity after bleaching is higher when HP is combined with thermal activation [4–9]. Diverging results once again have been published regarding the effect of power bleaching on the pulp [4–9]. Also for this topic there is a lack of in vivo studies and there are no studies evaluating long-term effects of HP exposure on dental pulp.

An intrapulpal temperature increase of 5.5°C is nowadays regarded as the threshold value, which should not be exceeded to avoid irreversible pulp damage [10]. It appears that temperature during light-activated bleaching is in general under control, especially due to the presence of a bleaching [4, 10].

## 2. Aim

At present, there is no review on the efficiency of laser activated bleaching and its effect on the tooth (enamel and pulp). The aim of this review is therefore to evaluate the influence of the temperature rise during laser bleaching on the pulp, the postoperative sensitivity, and eventual enamel alterations. The efficiency is evaluated on the basis of the colour change in vitro and in vivo.

## 3. Methods and Materials

The electronic literature search included the databases PubMed and Web of Science for manuscripts published with full journal reference from January 1950 to November 2014. All languages were accepted provided there was an abstract in English. The following MeSH terms and key words were used: “lasers” AND “tooth bleaching,” “lasers” AND “tooth discoloration,” “tooth bleaching” OR “teeth bleaching” AND (argon laser OR diode laser OR KTP laser OR Nd:YAG laser OR Er:YAG laser OR Er, Cr:YSGG laser OR carbon dioxide laser). Two reviewers (AD and BV) independently assessed abstracts and full-text articles. First the reviewers considered the abstracts as potentially relevant. Abstracts dealing with this topic but without access to full journal article were not taken into consideration. Case reports were included only when they exclusively reported observations which were not described in other publications. Then full articles were read. Both reviewers selected independently the same 71 full-text articles; that is, Cohen's kappa = 1.0.

## 4. Results

### 4.1. Temperature Rise in the Pulp

Taking into account the subject of the present review both power intensity and wavelength of the light used during the bleaching procedure must be taken into consideration [11]. An overview of the changes in temperature in the pulp during laser dental bleaching is given in Table 1.

#### 4.1.1. CO_2_ Laser (10,600 nm)

Luk et al. [12] reported that the use of a CO_2_ laser (10,600 nm) on teeth for bleaching purposes led to a temperature increase of 13.1 to 22.3°C with gel at the enamel surface and 6.9 to 16.6°C at the pulpal side of the dentine. Due to a lack of controlled clinical studies this wavelength was not approved for bleaching by the ADA [30]. At present this wavelength is no longer used for dental bleaching.

#### 4.1.2. Nd:YAG (1,064 nm)

Next to the CO_2_ laser, the highest temperature elevations in the pulp were registered with the Nd:YAG laser (1,064 nm) irrespective of the use of coloured bleaching gels (blue, red, and transparent) [13, 14].

#### 4.1.3. Diode Lasers

High power* diode lasers* (784–980 nm) are also known to be able to rise the pulpal temperature and should be used in combination with a bleaching gel. An overview of the reported data is given in Table 1.

Laser activation with a 830 nm* diode laser* (30 s, 3 W) without bleaching gel may result in a temperature increase of 16°C in the pulp chamber; when applying the gel during laser activation only 8.7°C temperature increase was recorded [10].

With a 915 nm* diode laser*, there was an increase of temperature with 26.7°C at 3 W-20 s and 12.2°C at 1.5 W-20 s [16]. In the same study temperature rise was lower after application of a bleaching gel; the decrease was product related: By White gel (By Dental, Pistoia, Italy) at 3 W resulted in a rise of 17.0°C and at 1.5 W of 7.6°C with Whiteness HP (FGM Produtos Odontológicos, Joinville, Brazil) it was +25.6°C at 3 W and +6.0°C at 1.5 W. The bleaching gel thus acts as a selective absorber near the dental surface, preventing light penetration into the internal tooth structure. Apparently the composition of the gel is also important as the gel layer was 2 mm thick in both investigations.

An increase of 2.61°C with a* diode laser* (810 nm) (4 W, 20 s) and 1.86°C with an Er:YAG (2,940 nm) (40 mJ, 10 Hz, 20 s) was registered by Sari et al. [17]. The temperature in the gel, however, was 6.21°C for the diode and 20.11°C for Er:YAG.

The increase in the pulp chamber temperature with a* diode laser* (830 nm) used at 1 W-30 s is below the critical temperature increase of 5.5°C that is nowadays regarded as the threshold value and which should not be exceeded to prevent irreversible pulp damage [18]. In the same study the diode laser at 2 W-30 s resulted in a temperature increase up to 6.8°C and 8.7°C with 3 W-30 s; the importance of use of the gel with appropriate thickness was emphasized by measurements of the temperature at the surface: 1 W resulted in 37°C, 2 W in 64.1°C, and 3 W in 86.3°C.

Similar findings were registered by Fornaini et al. [19] where an 808 nm* diode *at 2 W during 3 × 30 s resulted in heating of the gel up to 43.1°C and at 4 W-3 × 30 s up to 74.9°C.

A temperature increase of 2–8°C and 4–12°C was observed when a 960 nm* diode* laser was used to activate Opalescence Xtra (Ultradent Products, South Jordan, UT, USA) and Opus White (Opus Dent, London, UK) for 0.9 W-60 s and 2 W-30 s [20].

A mean increase of 11.75°C in the pulp was seen with an 810 nm* diode* used at 10 W-15 s [22].

With a hydrogen peroxide bleaching agent, the mean maximum pulpal temperature rise was 2.95°C for a LED, 3.76°C for a KTP laser (1 W-30 s), and 7.72°C for a 980 nm* diode laser* (0.8 W-30 s) [23].

With an output power of 1 W-30 s of an 810 nm* diode* laser, pulpal temperature increase was shown to be approximately 3°C with the Opus White gel (Opus Dent), whereas a TiO_2_ emulsion showed almost no temperature changes in the pulp [24].

A treatment protocol with intermittent irradiation of six times for 5 s, with 5 breaks in between, at a power setting of less than 1 W, with a 810 nm* diode laser* excluded thermal damage to the pulp, whereas the temperature at the surface was 5°C. Irradiation at 2 W with the same protocol resulted in a temperature elevation of 9.6°C at the surface and 2.8°C in the pulp chamber [25].

The* addition of colorants *may help to provide a better absorption of high power diode laser light in the bleaching gel and less transmission towards the pulp chamber. Pleffken et al. [26] demonstrated that a low-intensity red* diode laser* (660 nm) (50 mW-3 × 180 s) with a green-coloured bleach gel resulted in not more than a 2.3°C temperature elevation in the pulp chamber.

It is clear that power and type (wavelength) of the light source influence temperature variation. Studies have shown that near-infrared lasers could improve the inflammatory response of the pulpal tissue, reducing pulp damage and relieving pain after the bleaching process [31]. Its use would diminish patients' sensitivity complaints after the procedure. In this respect* LED devices *were associated with* diode lasers* emitting in the near-infrared. According to Carrasco et al. [27] 470/790 nm (40 mW-3 × 30 s) temperature is under control. Other studies also demonstrated negligible temperature changes: 470/784 nm (120 mW-5 × 40 s) [28], 470/795 nm (120 mW-3 × 60 s), and 530/795 nm (20 mW/3 × 60 s [29]). Moreover it appears that these combination types of light sources with low power density are not powerful enough to provide a better bleaching efficacy as compared to other light sources [20, 23, 32].

A comparison between different laser wavelengths by Dominguez et al. [14] used as follows: that is, three bleaching gels (transparent, with blue dye, with red dye, composition of dye mentioned) exposed during 3 times for 20 sec to the light source with a 9 min interval and an overall contact time of the gel with the tooth surface during 30 min, demonstrated the following temperature effects in decreasing order:* Nd:YAG* (1064 nm) (rise of 3.1°C in the pulp chamber) > halogen lamp (120 nm) >* low power diode* (675 nm) > low power LED (380–530 nm) >* 2*ω*Nd:YAG* (532 nm) >* Er:YAG* (2940 nm). These findings coincided with the findings of Torres et al. [28] (halogen versus diode 470/784 nm) and Carrasco et al. [27] (halogen versus diode 470/790 nm versus LED).

In a study of Klaric et al. [15] a comparison was made between ZOOM2 (350–400 nm) during 15 min, LED (405 nm) during 30 min, OLED (organic light emitting diode) (400–760 nm) during 30 min, and a* femtosecond laser* (770 nm) (Millenia, Spectra Physics, USA) during 30 min: ZOOM2 resulted in high temperature elevations (+15.4°C) in the pulp whereas elevations were +21.1°C without use of bleaching gel; the femtosecond laser focused: +15.7°C without gel and +8.7°C with gel; the femtosecond laser unfocused: +2.7°C without gel and +1.4°C unfocused. In this respect it has also to be mentioned that the mechanism of heat conversion depends directly on the tissue constituents and the irradiation wavelength used. It is known that the tooth absorption coefficient is lower for the wavelength range 400 < *λ* < 500 nm; thus scattering predominates over absorption at these wavelengths.

*Er:YAG (2,940 nm).* In a study of Kivanç et al. [16] temperature increase in the pulp was neglectable. A very low temperature rise of 1.86°C was registered by Sari et al. [17].

*KTP (532 nm).* Using the green light of the KTP to irradiate a red coloured bleaching gel resulted in a temperature of 32°C at 2 W during 30 sec and 45.1°C at 4 W during 30 sec [19].

With a hydrogen peroxide bleaching agent, the mean maximum pulpal temperature rise was 2.95°C for a LED, 3.76°C for a KTP laser, and 7.72°C for a diode laser [23].

### 4.2. Influence on the Characteristics and Material Properties of the Teeth

The aim of a bleaching procedure is to bleach the tooth without morphological and chemical changes. However, side effects after power bleaching in the enamel such as changes in microhardness, the presence of porosities, changes in surface roughness, a reduction in fracture toughness, alteration of the calcium/phosphate ratio, erosion, decrease in abrasion resistance, and the formation of depressions were reported. Weakening of enamel structure by oxidation of organic or inorganic elements is considered to be the main cause [33].

#### 4.2.1. Morphological Analysis

Morphological analysis showed slight changes with the diode laser (970 nm) and the LED/laser (467 nm/790 nm) [34]. Surface effects were unrelated to the pH of the high concentration HP bleaching gels with laser activation and referred more to a better or lesser absorption of the laser light by the bleaching gel. Chromophores and the use of TiO_2_ appeared to be favourable for the maintenance of an intact tooth surface [25]. No significant effects on the morphology of the enamel surface after laser bleaching with diode laser, KTP, Nd:YAG, and Er:YAG were observed by Dominguez et al. [14].

#### 4.2.2. Mineral Content

FT-RS results showed a decreased mineral content after bleaching procedures with and without light activation and with 35% HP-based bleaching agents. The use of a LED/laser (470 nm/810–830 nm) resulted in comparable calcium loss as compared to the non-light-activated bleach gels for 2 of 3 brands. Exposing Pola Office (Southern Dental Industries, Sao Paulo, SP, Brazil) to the LED/laser did not result in a significant calcium loss [35]. An explanation for this difference was not given.

No significant differences in levels of calcium and phosphorus were seen after 470/830 nm LED/laser bleaching [36].

In a study of Cesar et al. [37] with 35% HP-based bleaching agents activated with a LED/laser (465.5 nm/790 nm), FT-Raman spectroscopy data showed no significant chemical changes in the inorganic components for the tested groups. Carbonate and phosphate area peaks were not significantly changed. Whiteness HP Maxx (FGM Produtos Odontológicos Ltda., Santa Catarina, Brazil) and Opalescence Xtra (Ultradent Products) were also tested in the study of Berger et al. [35]. There was a significant reduction of the dental organics associated with type I collagen vibration only in the group of Whiteform-Perox Red gel (Formula & Acao, Sao Paulo, SP, Brazil). This means that there is a difference between both studies. Total contact time of the gels was identical, that is, 3 consecutive gel applications for 10 min. Irradiation protocols, however, differed: in Berger et al. [35], there was a bleaching gel left on third molars undisturbed for 1 min and then irradiated for 2 min; the light irradiation was repeated 3 times with a 1 min interval between radiations; in Cesar et al. [37] there was photoactivation of the gel for 30 sec for a total of 10 min of application on bovine teeth. Moreover, similar differences in findings were also observed with non-light-activated high concentration HP bleaching gels. Whether the differences found for the present two studies are related just to the bleaching protocol is not clear yet; in fact differences in oxidizing potential (stronger), stronger concentrations, longer treatment times, and lower pH of bleaching gels could be responsible for the changes found in the studies [37].

In a study with bovine teeth using a low power diode laser (740 nm, 300 mW power) it was seen that the enamel crystallinity was dramatically decreased by a bleaching treatment without laser irradiation. However, crystallinity increased as laser irradiation time increased. It was concluded that professional bleaching treatment with HP combined with a diode laser irradiation not only improves the bleaching effect but also protects against the change of enamel structure compared with the bleaching treatment without laser irradiation [38].

TEM analysis showed the formation of a new phase 2 *μ*m thick layer. The assumption was also made that the chemical property of the bleaching gel could have been changed through exposure to laser irradiation. It can also be that this phenomenon accounts for bovine teeth where the enamel contains significantly more interprismatic organic material compared to human enamel even though its structure and compositions are very similar to those of human enamel.

#### 4.2.3. Microhardness

The microhardness test is suitable for determining small changes in surface that demonstrated the effect of bleaching products on enamel [39].

A comparison between argon laser (488 nm, 200 mW, 30 sec irradiation and 4-minute intervals during 40 min) and halogen lamp-based photopolymerizer (2 min and 240 mW, and 4-minute intervals during 40 min) did not result in differences with the control group using 35% and 37% CP [37].

Zhang et al. [23] showed no differences between the control (35% HP) and KTP (1 W, 30 sec, energy density (ED) 13.33 J/cm^2^), diode 980 nm (0.8 W, 30 sec, ED: 13.33 J/cm^2^), and blue LED composite curing lamp (470 nm, 30 s, ED: 12.6 J/cm^2^) experimental groups. Diode laser (830 nm) irradiation (3 times, 30 sec irradiation at 1.4 W of newly placed) of the 35% HP gel associated or not with ACP did not interfere with microhardness [40].

Reduction in microhardness was found after bleaching with a LED/laser (470/830 nm, light intensity of 200 mW, 1 min laser activation of the gel, followed by 2-minute rest; this procedure was repeated 3 times), which recovered to baseline values after 1 week of immersion in artificial saliva [36].

#### 4.2.4. Enamel Permeability

Higher permeability of the enamel surface after a bleaching procedure with a LED/laser (470–790 nm) and QTH light as compared to a control (35% HP) was reported; there were no significant differences between the two bleach protocols [41].

Bleaching with a 470/830 nm LED/laser did not show any statistical difference with baseline with regard to dye penetration [36].

#### 4.2.5. Caries Susceptibility of Bleached Enamel

In-office laser bleaching with a LED/laser (830 nm) does not result in a higher susceptibility for caries lesions [42].

#### 4.2.6. Fracture Strength

Araujo et al. [43] showed that a LED/laser (465.5 nm/790 nm) did not influence the fracture strength of enamel after light-activated bleaching.

#### 4.2.7. Bonding to Bleached Enamel

Bonding to intracoronally light-activated bleached dentine should be performed at least 10 days after a bleaching procedure with a LED/laser (465.5 nm/790 nm) [44]. A time interval of 2 to 3 weeks was advocated for applying silorane-based composite restorations of methacrylate based composites after bleaching with an 815 nm diode laser [45]. A week interval after bleaching with an 815 nm diode and 430–490 nm blue LED showed statistically significantly lower shear bond values as compared to the control and bleaching with QTH light (380–520 nm) [44]. The failure mode in this latter study was adhesive for the diode laser (80%) and the LED (70%); for both the control group and the QTH lamp the failure mode was mixed (adhesive and cohesive) (70%).

### 4.3. Hypersensitivity

#### 4.3.1. Diodes and LED/Lasers (Diodes)

Sensitivity is described by some as common with the diode laser. Bleaching with a diode laser (810 nm, 35% HP) just reached the level that can be tolerated by the patient [46]. Comparing a diode laser (810 nm, 37% HP) with PAC activation (400–490 nm, 35% HP), LED activation (400–500 nm, 38% HP) and no light activation (38% HP) resulted in the lowest sensitivity for the diode laser [47].

In the study of Kossatz et al. [48] 53.3% of the participants had sensitivity even 24 hours after laser bleaching with a LED/laser unit (470 nm/830 nm, 35% HP) with a protocol of gel activation during 1 min, leaving the gel undisturbed during 2 min and repeating this protocol 3 times and the in-office bleaching agent was refreshed every 15 minutes during a 45-minute application period. Immediate sensitivity was also scored in the study of Mondelli et al. [47] with a LED/laser (470 nm/810 nm, 35% HP). Sensitivity decreased after 24 hours to return to normal after 7 days. There were no differences between in-office gels (light- and non-light-activated).

An increased expression of substance P was seen when a LED laser (470 nm, 35% HP) was used [49].

More recent studies demonstrated that sensitivity was generated independently of the light sources used: Almeida et al. [50] with a LED/laser at 425–480/810 nm, Martin et al. [51] with a LED/laser at 450/808 nm, and Moncada et al. [52] with a LED/laser at 425–480/808–830 nm. The latter two studies demonstrated a higher impact of the increase in concentration of bleaching agents on tooth sensitivity; treatment with carbamide peroxide generated also lower sensitivity than treatment with HP independently of the light sources.

A comparison between all these studies is difficult and impossible because each investigation is different, that is, different protocols. Moreover, complete basic information, that is, power settings, gel thickness, distance between gel, and light source, is not provided in the listed studies.

#### 4.3.2. Nd:YAG

When using the Nd:YAG laser (1,064 nm, 35% HP) for laser bleaching at 4 W, 10 Hz, 320 *μ*s pulse duration, and an energy density of 1.4 J/cm^2^ in association with a red coloured gel, no enhancement of the bleaching success was found [52]. There was no reduction in hypersensitivity, as can be seen when Nd:YAG is used for the treatment of dentinal hypersensitivity. Only 20% of the patients in this study had a pain-free treatment with the Nd:YAG laser. The remaining patients felt the development of warmth to the point of pain, even a few days after laser treatment. The authors concluded to query the appliance of Nd:YAG laser irradiation for in-office bleaching.

### 4.4. Laser Bleaching: Tooth Colour Change and Efficiency

#### 4.4.1. Colour Change In Vitro

A comparison based on analysis of photoreflectance spectra between the use of an argon laser (488 nm) and halogen lamp with 35% and 37% CPO gave better results for the 35% CPO gel. Halogen was as effective as argon laser with 35% CPO; argon was more effective than halogen for the 37% CPO [32]. A comparison between LED/diode laser (450–500 nm/830 nm), argon (488 nm), PAC (440–550 nm), and halogen (350–500 nm) showed better results for a 35% HP than for 37% CBP. A decrease in reflectance values was seen after 30 days; no difference was observed in bleaching efficiency between activated and nonactivated bleaching gels with high HP concentrations [53].

In a study comparing KTP (532 nm, 35% HP) with a diode laser (810 nm, 38% HP, 37% HP, and 35% HP) [25] improved changes in brightness of up to ten steps on the Vitapan classical shade guide were detected. Prerequisites, however, were a perfect match of the chosen wavelength and the bleaching gel. A neutral and basic pH of the bleaching gel is also advantageous. The higher bleaching power of KTP as compared to an 808 nm diode laser was confirmed by Fornaini et al. [19].

Diode laser activation (808 nm, 35% HP) of the bleaching agent was not more effective than the halogen lamp for bleaching root canal treated primary molars [54], safer for* T*° development. Activation of a 35% HP bleaching gel by diode laser (830 nm, 35% HP) as well as a xenon halogen light, a plasma arc lamp, and halogen light did not differ in result from the use of the same gel without light activation [55]. A comparison between a 980 nm diode laser and a xenon arc lamp (430–500 nm) used with a 35% HP bleaching gel showed that there was an increase in colour saturation (Δ*C* ^*^) of 3–32% and a change in whiteness (Δ*L* ^*^) of 0–8% [14]. The highest efficacy was achieved with the diode laser at 2 W, the lowest with the diode laser at 0.9 W. However, due to the risk of higher temperature development, the authors considered the xenon lamp as the safest. A comparison between a diode laser (808 nm) and LED (471 nm) demonstrated significant comparable change in chroma for the two 35% HP bleach gels investigated and the light sources. There was also a significant change in lightness for all test conditions, but the diode scored significantly best with the Whiteness HP bleaching agents (FGM Produtos Odontológicos, Joinville, Brazil) than with Opalescence Xtra (Ultradent Products) [21].

A comparison between different laser wavelengths by Dominguez et al. [14] demonstrated that the source of irradiation was more relevant than the bleaching agent for efficient tooth bleaching. They exposed three 35% HP bleaching gels (transparent, with blue dye, and with red dye, composition of dye mentioned) during 3 times for 20 sec to the light source with a 9 min interval; contact time of the gel with the tooth surface was 30 min. LED (380–530 nm, low power), halogen lamp (120 nm), and diode (675 nm, low power) produced greater colour changes than the rest of the light sources: Nd:YAG (1064 nm), Er:YAG (2940 nm), and 2*ω*Nd:YAG (532 nm). The mean improvement in tooth whiteness with the latter three wavelengths is in the same order as without photoactivation. It is thus the question if these wavelengths are really suited for bleaching gel activation. These findings differ from other studies where the effect of an Nd:YAG was comparable with a halogen light [56], and the effect of KTP was better as compared to diode (980 nm) and blue LED (470 nm) [23], but these chromophores were chosen as a function of the wavelength used (Nd:YAG: Q-switch dye with maximum absorption at 1051 nm) (KTP: sulphorhodamine B with maximum absorption at 542.8 nm).

#### 4.4.2. Clinical Efficacy

When the Nd:YAG laser (35% HP) was used for bleaching, Strobl et al. [57] found no supportive influence of the laser radiation on the bleaching. The authors registered a change in the colour of the bleach gel after laser activation, being a result of the increased formation of chemicals radicals, but could not explain why this does not translate in improved clinical result.

The use of a low-intensity red diode laser (660 nm, 35% HP) with a green-coloured bleach gel resulted in a change of colour (Δ*E* was increased from 5.4 to 7.2 after 1 week) [26].

Overall shade change values recorded by spectrophotometer reading expressed as Δ*L*, Δ*a*, Δ*b*, and Δ*E* were significantly higher for diode laser (810 nm, 37% HP) bleaching than PAC activation (400–490 nm, 35% HP), LED activation (400–500 nm, 38% HP), and no light activation (38% HP), although shade guide evaluations did not exhibit any differences [58]. One session of 20 min of in-office bleaching with or LED (470 nm, 35% HP) (3 min irradiation to each group of 3 teeth) or diode laser (808 nm, 35% HP) (30 sec irradiation per tooth) as initiator followed by 10% CP home-bleaching during 7 days was not more effective than 10% CP home-bleaching alone during 14 days [59]. Bleaching with an 841 nm diode laser and 35% HP showed greater shade improvement for teeth with hue A shade than those with hue C and D. The bleaching process is better in younger patients and gender is not a factor that affects the bleaching process [46].

LED/laser at 470 nm (one wavelength mentioned) did not show any improvement in bleaching result for the treatment of vital teeth as compared to halogen light, LED, and non-light-activated 35% HP. All treatments resulted in an increase of Δ*E* (best score for the non-light-activated protocol), which was maintained for 1 month and then dropped at 6 months (from on average 8 at 1 month to 7.7 at 6 months for the light-activated systems and from 9.8 to 8.8 for the non-light-activated group) [60] but also meaning that there was only a slight colour rebound. LED/laser (470 nm/808 nm) did not improve the in-office bleaching results with 35% HP as compared to QTH (quartz-tungsten-halogen) light and at home bleaching with 10% CPO [50]. A change in colour was registered for all protocols, which was maintained over a 6-month period [50]. In another investigation LED/laser (450–500 nm/830 nm, 35% HP) did not improve the bleaching effectiveness during any phase of the study [27] as compared to two different LEDs (450–500 nm at 164 mW and 430–490 nm at 88 mW, with 35% HP) and a halogen lamp (350–500 nm at 470 mW, 35% HP) and additional sessions did not improve the results obtained in the first session. Change of color was registered for all systems. In a study by Mondelli et al. [47] in-office bleaching (35% and 38% HP) with and without activation with a LED/laser (470 nm/810 nm) was compared with home bleaching (15% CPO). All techniques and bleaching agents were effective. There was no difference in Δ*E* between non-light- and light-activated in-office treatment. The initial increase in Δ*E* decreased over a time period of 24 months (from on average 7.8 to 2 for the high concentration HP, from 9.8 to 3.3 for the home bleaching procedure with 15% CPO).

Visible green light KTP laser (532 nm, 35% HP) combined with sulphorhodamine B-photosensitizer bleaching gel activated for 30 sec at 1 W provided a clinically useful improvement in tooth shade in teeth with tetracycline discolorations [61, 62]. KTP was more efficient than a 810 nm diode laser for the removal of discolourations due to red fruits, tea, and coffee [63]. KTP is more efficient for tetracycline discolouration than a high powered green LED for the bleaching of tetracycline-stained dentine [64].

## 5. Discussion

Light sources are marketed with the idea that light plays a significant role in tooth bleaching as catalyst for the ionization of HP in the bleaching gel and increasing the bleaching effect. Studies on light sources with incoherent light sources have produced contradictory results, but the following conclusions were drawn on the basis of a systematic review: (1) both light-activated and non-light-activated systems showed similar immediate and short-term bleaching effects when high concentrations of HP (25–35%) were used as bleaching gel; (2) there is limited evidence that a light-activated system produced better immediate bleaching efficacy than when non-light-activated systems with a lower concentration of HP (15–20%) were used [9].

Two key factors determining overall tooth bleaching efficacy from peroxide containing gels are the concentration of the HP and the duration of application.

For as far as the specific topic of laser activated bleaching is concerned, contradictory results are found as was also seen with conventional bleaching procedure using high hydrogen peroxide concentrations. In addition, the number of laser activated bleaching studies is limited as compared to the literature on light-activated bleaching. Comparisons between the effects of different wavelengths are difficult to make: (1) for laser bleaching absorption in the bleaching gel is aimed to drive the ionization of the HP; this depends on the specific wavelength needed to directly photolyze or photooxidate the chromophores in the dentine; (2) the chosen wavelength has to coincide with the absorption peak of chromophores or photocatalysers in the bleaching gel (if present) in order to catalyse the ionisation of the hydrogen peroxide and to drive the photolysis; (3) there is the heterogeneity of the heating temperature of the gel, that is, the photothermal effect which is even so influenced not only by the wavelength, but also by the specific power settings; (4) because of the previously mentioned heterogeneity of the laser settings, seen when a specific laser wavelength is considered, the bleaching gel must be developed taking into account the specific laser wavelength; (5) in addition, power density or energy density (fluence) of the laser beam is important; temporal characteristics of the laser beam are to be considered such as continuous versus pulsed delivery and consequently the pulse rate and the pulse duration; other variables that relate to differences in the method of energy transfer such as contact versus noncontact delivery mode, focused versus unfocused, and beam diameter have also to be considered. Last but not least there are the differences in the exposure time of the gel to the laser light and the specific bleaching protocol (e.g., one exposure or consecutive exposures of a fresh bleaching gel do also contribute to the heterogeneous data).

In general, laser bleaching is performed with a hand piece or a fibre in noncontact mode, unfocused, and with continuous emission. Regarding the power or energy, high power lasers are generally used, except when bleaching is performed with the argon laser (488 or 514.5 nm) or with a 660, 675, or 740 nm diode laser.

All studies selected for this survey on laser bleaching have in common the fact that a high HP concentrated bleaching gel is used (35 to 38% HP and 35 or 37% CP, i.e., 12 to 13% HP). None of the clinical studies used low concentrations of HP. For HP concentrations of 6%, it is known that light activation produced better immediate bleaching effects [9]. The EU Council Directive 2011/84/EU of September 20, 2011 [65], restricts the use of bleaching and bleaching products: only dentists may use products for tooth bleaching and only bleaching products that contain or release between 0.1% and 6% HP and products for tooth bleaching and bleaching that contain or release up to 0.1% HP are available as over-the-counter products. Products with HP concentrations over 6% are prohibited as cosmetics. This clearly means (1) that products containing or releasing more than 6% are prohibited for dental bleaching and (2) that dental bleaching is not considered as a medical action but only as cosmetical and hence nonhealing procedure. Information on laser activation of bleaching products up to 6% with lasers has not been published.

A number of wavelengths can be considered as not recommended for laser bleaching: Nd:YAG (1,064 nm), Er:YAG (2,940 nm), and CO_2_ (10,600 nm). The effect of these laser wavelengths is purely based on heating of the bleaching gel (Nd:YAG) or should only be restricted to heating of the bleaching gel (Er:YAG and CO_2_: care has to be taken not to remove tooth substance with Er:YAG and CO_2_ because both wavelengths are well absorbed by water and hydroxylapatite which might result in superficial ablation of tooth substance). Although the CO_2_-laser received an FDA approval for bleaching, the ADA soon after recommended not to use this wavelength for bleaching.

From all bleaching wavelengths the diode wavelengths have been most extensively investigated. A large range of diode wavelengths are used as laser bleaching wavelengths. These near-infrared lasers are used at low power or at high power. Both low power and high power diode lasers do not result in an enhanced bleaching efficacy when compared to non-light-activated bleaching with high HP concentrations. The question is even if low power diodes aid in the activation of the bleaching gel. Care, however, has to be taken with the high power diodes so as not to heat the bleaching gel at a level at which thermal damage of the pulp might occur.

Another key factor to increase the rate of the chemical reaction is to increase the temperature, where a rise of 10°C can double the reaction rate. On the one hand the thickness of the bleaching gel layer is important to ensure that the laser light can pass through this layer. The distance between the handpiece or fibre end and the gel is important when the energy is considered. Laser interaction is not limited to the gel alone and laser light has also to interact with the discolouration in the tooth.

Adding chromophores, chosen in accordance to the absorption peak of the gel, acts as a selective absorber near the dental surface, preventing light penetration into the internal tooth structure. The colour of the gel is important as it influences the final temperature, since different light sources have different emission wavelengths and the absorption peak changes following gel colour. Also here the question is if the dyes added for photoactivation of the gel with diode lasers are helpful in activating the bleaching gel. With high power diode lasers, irrespective of the thickness of the bleaching gel, care has to be taken still so as not to extensively dehydrate the enamel due to the temperature effect.

Recently LED devices were associated with diode lasers emitting in the near-infrared, which, with appropriate energy density, are being used to desensitize the teeth under bleaching [24, 66]. These studies demonstrated that near-infrared lasers could reduce the inflammatory response of the pulpal tissue, reducing pulp damage and relieving pain after the bleaching process. The use of these devices (so-called LED lasers), however, did not result in any increased bleaching efficacy. Thus the question is to what extent these low power diodes are of help in the bleaching process.

Light-activated systems were found to increase the occurrence of severity of tooth sensitivity [9]. The light source itself can increase pulpal temperature leading to increased tooth sensitivity [66]. The latter was also encountered with diode lasers [46, 48] and Nd:YAG [13, 14, 52]. For both wavelengths laser light is transmitted through the bleaching gel in combination with a heating of the gel, irrespective of the thickness of the gel leading to tooth sensitivity [10, 14, 16, 19]. An additional explanation is also that laser activated bleaching may increase the expression of substance P in the human dental pulp [58, 67].

Taking into account all different wavelengths used for laser activated bleaching, the KTP laser when used at appropriate settings and combined with the red coloured bleaching gels (Smart Bleach, SBI) has been shown to be one of the best options for photoactivated dental bleaching. Walsh [68] demonstrated a higher bleaching effect with KTP than with a diode laser based on DOTCAM analysis, a result which was also confirmed in other studies [19, 23, 69]. Its efficacy was also demonstrated for the bleaching of tetracycline discoloured teeth [61]. Temperature elevation in the pulp chamber was also under control when appropriate settings were used in conjunction with a red coloured gel (containing sulphorhodamine B as a chromophore) [19, 24]. The safety of the procedure was demonstrated by an unaltered enamel surface after KTP laser bleaching [25]; no significant differences in the enamel microhardness pre- and posttreatment [68] and no changes in the compositional structure of dentin surfaces were found [69]. Occasional mild postoperative sensitivity was seen during the 12 h following the procedure as radicals are neutralized by catalase and other pulpal enzymes [1, 68]. Catalase had been found to protect the dental pulp during vital bleaching procedures [70]. A catalase application was demonstrated to eliminate residual hydrogen peroxide during non-vital bleaching procedures [71].

## 6. Conclusions

1. It is difficult to draw conclusions for laser bleaching on efficiency and efficacy from the present-day literature because of the difference in concentrations in hydrogen peroxide used, the difference in wavelengths of lasers (especially the diodes) used, the difference in laser settings and protocols used, and differences in bleaching gels used with or without photocatalyst.
2. Comparative studies evaluating bleaching techniques with high concentrations of hydrogen peroxide and with or without the use of light activation resulted in enhanced lightening. Most often comparable results were found irrespective of light exposure.
3. No long-term evaluations for laser enhanced bleaching procedures are available.
4. Based on the limited number of investigations, at present, only one particular wavelength appears to be able to perform direct photobleaching (or photooxidation), that is, KTP (532 nm). When KTP is used in combination with a bleaching gel containing a chromophore (sulphorhodamine) allowing the absorption of the laser light, photodynamic reactions can be induced (photochemical activation of the gel with limited photothermal activation). This combination of wavelength and specifically dyed bleaching gel also allows for safe bleaching (no damage of the enamel, no heating of the pulp) when the guidelines of the manufacturer are followed.
5. At present a number of wavelengths are not recommended for laser bleaching: Nd:YAG, Er:YAG, and CO_2_. Combination devices consisting of LED-diode laser do not result in enhanced lightening and are in fact not effective. When using high power diode lasers for bleaching care has to be taken so as not to overheat the pulp. Also diode lasers are not really advocated for laser bleaching except when the wavelength is used in combination with a bleaching gel containing wavelength specific absorbers.
6. With the exception of KTP used in combination with a gel with a specifically red coloured light absorber (sulphorhodamine B) for the green light (532 nm), laser activated bleaching is solely based on heating of the bleaching gel.
7. All studies have been conducted with high concentrated hydrogen peroxide gels. This means that the soft tissues have to be thoroughly protected during the in-office power bleaching procedure. No studies were conducted to investigate the safety of laser bleaching procedures on the soft tissues adjacent to the laser activated bleaching gel.

## 7. Recommendations for Future Investigations

Three factors are to be considered when using a light source and should be mentioned in the studies: light intensity, spectral distribution, and irradiation time. Since the total energy depends on light intensity and irradiation time, light curing units with high intensity may allow a reduction in irradiation time. Second generation LEDs present higher power than first generation LEDs. Further research is needed to evaluate if high power (narrow band) LEDs can be used for light-activated bleaching. With the price of a number of laser devices in mind, this technology might be of interest for the activation of bleaching gels.

An important relationship exists among gel colour, laser wavelength, thermal transmission, and clinical efficacy, but not between gel temperature, shade change, and HP concentration. In this respect the use of absorbing substances to increase the radiation absorption (and the temperature in the gel) is known. With the use of TiO_2_ it has been demonstrated that there is another way to improve dental bleaching without the risk of damaging the pulp. Hence the composition of the gel with the absorbers and additional compounds (agents enabling to catalyse the redox reaction) should also be given in detail.

## Figures and Tables

**Table 1:** Increase in temperature (°C) in the pulp (without or with gel application) after exposure to laser light.

Authors	Wavelength	Settings	Bleaching gel	Result/temperature
Luk et al., 2004 [12]	10,600 nm (CO_2_)	600 mW 6 × 30 sec/180 sec interval GT: 2 mm *D*: 1 to 2 mm	Opalescence Extra Quick White Star Brite Nypro Gold	Pulp without and with gel +13.34 +9.75 +10.73 +6.93 +13.08 +7.98 +22.26 +16.55

Michida et al., 2009 [13]	1,064 nm (Nd:YAG)	600 mJ, 10 Hz (0.6 W) 20 sec GT: 0.5 to 1 mm *D*: 5 mm	Whiteness HP	Pulp with gel +4.33

Dominguez et al., 2011 [14]	1,064 nm (Nd:YAG)	75 mJ, 10 Hz 3 × 20 sec GT: 2 mm *D*: 2 mm	Ena White Power Opalescence Endo Q White	Pulp with gel +3.1 +2.5 +2.9
	2,940 nm (Er:YAG)	40 mJ, 10 Hz 3 × 20 sec GT: 2 mm *D*: 2 mm	Ena White Power Opalescence Endo Q White	Pulp with gel +0.2 0 0
	532 nm (diode)	1.6 mJ, 15 Hz 3 × 20 sec GT: 2 mm *D*: 2 mm	Ena White Power Opalescence Endo Q White	Pulp with gel +0.25 0 0
	675 nm (diode)	200 mW 3 × 20 sec GT: 2 mm *D*: 2 mm	Ena White Power Opalescence Endo Q White	Pulp with gel +2.6 +1.3 +1.3

Klaric et al., 2013 [15]	770 nm (femtosecond diode)	800 mW, 15 min Unfocused GT: ? *D*: ?	Without gel ZOOM 2 Boost Vivastyle 30 Vivastyle 16 Vivastyle 10	Enamel surface Pulp +3.2 +2.7 +2.1 +1.4 +2.0 +1.4 +2.1 +1.4 +2.1 +1.4 +2.2 +1.4
		800 mW, 15 min Focused GT: ? *D*: ?	Without gel ZOOM 2 Boost Vivastyle 30 Vivastyle 16 Vivastyle 10	Enamel surface Pulp +16.8 +15.7 +9.4 +8.7 +9.6 +8.8 +9.5 +8.7 +9.7 +8.8 +9.4 +8.6

Sulieman et al., 2005a [10]	830 nm (diode)	3 W, 30 sec GT: 2 mm *D*: just above the surface of the gel	Opus Mix bleaching powder + 35% HP liquid	Pulp without and with gel +16 +8.7

Kivanç et al., 2012 [16]	915 nm (diode)	3 W, 20 sec GT: 2 mm *D*: 10 mm	By White (BW) Whiteness HP (WHP)	Pulp without and with gel +26.7 BW +17 * * WPH +25.6
		1.5 W, 20 sec GT: 2 mm *D*: 10 mm		Pulp without and with gel +12.2 BW +7.6 * * * *WPH +6

Sari et al. [17], Epub 2013	810 nm (diode)	4 W, 20 sec GT: *D*:	Whiteness HP	Pulp with gel +2.61
	2940 nm (Nd:YAG)	40 mJ, 10 Hz, 20 sec GT: *D*:		Pulp with gel +1.86

Sulieman et al., 2006 [18]	830 nm (diode)	30 sec 3 W 2 W 1 W GT: 2 mm *D*: just above the surface of the gel	Opus Mix bleaching powder + 35% HP liquid	Surface Pulp without and * * with gel 86.3 +11.6 +8.7 64.1 +7.7 +6.8 37 +5.23 +5.5

Fornaini et al., 2013 [19]	808 nm (diode)	3 × 30 sec—rest time 2 min 2 W 4 W GT: *D*:	48 g HP solution + 29 g carbopol (HP concentration 27 to 35%)	Peak gel temperature 43.1 74.9
	532 nm (KTP)	3 × 30 sec—rest time 2 min 2 W 4 W GT: *D*:		Peak gel temperature 32 45.1

Wetter et al., 2004 [20, 21]	960 nm (diode)	0.9 W, 60 sec GT: ? *D*: 2 mm	Opalescence Xtra (OPX) Opus White (OW)	Pulp with gel OPX +8 OW comparable (8)
		2 W, 30 sec GT: ? *D*: 2 mm		Pulp with gel OPX +12 OW comparable (8)

Eldeniz et al., 2005 [22]	810 nm (diode)	10 W, 15 sec GT: OPX?—QS: 1 mm *D*: ?	Opalescence Xtra (OPX) Quasar Brite (QS)	Pulp with gel +11.75

Zhang et al., 2007 [23]	980 nm (diode)	0.8 W, 30 sec GT: 1 mm *D*: 1 mm	Hi Lite	Pulp with gel +7.72
	532 nm (KTP)	1 W, 30 sec GT: 1 mm *D*: 1 mm		Pulp with gel 3.76

Verheyen et al., 2006 [24]	810 nm (diode)	1 W, 30 sec GT: ? *D*: ?	Opus White (OW) Ti-O2 gel (TO)	Pulp with gel OW 3 TO 0

Goharkhay et al., 2009 [25]	810 nm (diode)	1 W, 60 sec 2 W, 60 sec GT: ? *D*: 6 mm	Opalescence Xtra Boost	Pulp without and with gel +5 0 +9.6 +2.8

Pleffken et al., 2012 [26]	660 nm (diode)	50 mW, 3 × 160 sec GT: ? *D*: 2 mm	35% experimental HP gel	In the gel Pulp with gel +2.29 +1.8

Carrasco et al., 2008 [27]	LED-laser 470/790 nm	40 mW, 3 × 30 sec GT: 1 mm *D*: 5 mm	Whiteness HP	Pulp without and with gel +0.35 +0.33

Torres et al., 2008 [28]	LED-laser 470/784 nm	120 mW 5 × 40 secc GT: 2 mm *D*: 5 mm	Whiteform Perox Red	Critical temperature rise of 5.5°C not reached

Coutinho et al., 2009 [29]	LED/laser 470/795 nm LED/laser 530/795 nm	120 mW, 3 × 60 sec 20 mW, 3 × 60 sec	Whiteness HP	Pulp with gel Incisor +3.1 Canine +2.5 Premolar +1.9 Incisor +0.6 Canine +0.2 Premolar +0.3

GT: gel thickness; *D*: distance between light source and the bleaching gel.
